# Subtrochanteric osteotomy for femoral mal-torsion through a surgical dislocation approach

**DOI:** 10.1093/jhps/hnv011

**Published:** 2015-02-18

**Authors:** Atul F. Kamath, Reinhold Ganz, Hong Zhang, Guido Grappiolo, Michael Leunig

**Affiliations:** 1. Department of Orthopaedic Surgery, University of Pennsylvania, Philadelphia, PA, USA; 2. University of Berne, Berne, Switzerland; 3. Department of Orthopaedic Surgery, The 1st Affiliated Hospital of PLA General Hospital of CPLA, Beijing, China; 4. Santa Corona Hospital, Livio Sciutto Onlus Foundation for Orthopedic Biomedical Research, Via XXV Aprile, 38 17027, Pietra Ligure SV, Italy; 5. Department of Orthopaedic Surgery, Schulthess Klinik, Zürich, Switzerland

## Abstract

Missed torsional femur deformities may contribute to reasons for failure after open and more likely arthroscopic hip preservation surgery. A number of surgical approaches have been described for addressing torsion abnormalities. This report describes a subtrochanteric osteotomy technique in a consecutive series of patients with complex hip pathologies, for which intertrochanteric osteotomy is not suitable and precise derotation is required. Subtrochanteric derotation was performed, always in combination with a surgical hip dislocation, in accordance with the authors’ preferred technique. Before osteotomy, a localized decortication was executed. Application of a 4.5-mm broad or narrow plate was undertaken with dynamic compression of the osteotomy. Twenty-eight consecutive subtrochanteric derotational osteotomies were performed in 26 patients. Twenty-one females and five males were treated at an average age of 21.4 years (range, 12–43). Underlying diagnoses included dysplasia, arthrogryposis, cerebral palsy, Down’s syndrome, instability and impingement. The decision to perform derotation was for antetorsion over 20° or less than 0° (retrotorsion). Patients were followed clinically and radiographically till final follow-up. All patients went on to successful osteotomy union. There were two initial failures: one delayed union prompting revision fixation in a chronic smoker and one plate failure due to self-accelerated weight-bearing in a patient status post successful contralateral derotational osteotomy. Rotational deformity of the femur must be considered in the patient undergoing hip preservation surgery. This technique of subtrochanteric derotational osteotomy, with adjunctive surgical hip dislocation, is applicable and reproducible in the setting of complex hip pathologies. **Level of evidence**: IV, case series.

## INTRODUCTION

Femoral torsional abnormalities are common [1, 2] in patients presenting with hip and groin pain. Our understanding of this phenomenon is limited; however, some literature suggests that unrecognized torsional deformities may compromise the results of open, and perhaps more commonly, arthroscopic preservation surgery of the hip [3]. Missed pathologic torsion may contribute to components of residual pain, poor functional outcomes and reason for failure of treatments related to hip impingement, instability and dysplasia [3, 4]. Torsional problems may accompany a number of hip conditions, including coxa vara, Legg–Calvé–Perthes disease, sequelae of cerebral palsy and slipped capital femoral epiphysis [5, 6]. The role of torsional issues in the ultimate development of hip osteoarthritis is a topic of active debate [2, 7–10].

Increased awareness has been given towards identifying femoral torsional problems. More than one type of deformity is usually present in those patients undergoing evaluation for hip pain [11]. Torsional components may contribute to intra-articular and extra-articular impingement [12], as well as instability [13] and tendency to traumatic posterior dislocation [14, 15]. Improvements in advanced cross-sectional diagnostic imaging, such as standardized protocols for computed tomography (CT) [16] and magnetic resonance imaging [8, 17], have proven useful adjuncts to clinical examination for more accurate measurement of rotational anatomy.

In the general algorithm for management of complex hip deformities, careful attention must be made to the utility of femoral deformity correction. In the sequential management of complex pathology, femoral osteotomy may precede and, depending on the specific clinical scenario, may obviate the need for additional pelvic correction [18]. Although a number of treatment strategies have been described for addressing femoral mal-torsion, including traditional blade-plate application [19, 20], both the location of osteotomy and the type of procedure have implications for effective reconstruction. Lower extremity rotational alignment has been corrected in various anatomic locations, including intertrochanteric, subtrochanteric, diaphyseal and supracondylar regions [5, 6, 19, 21–23] and with different fixation techniques and devices [5, 6, 19, 21, 24–28].

The rate of complications in the literature [25–27] prompts the continued search for improved surgical techniques. Although the role of abnormal torsion in causing later degenerative changes is not completely elucidated, we support the correction of intra- and extra-articular causes of impingement that might contribute to continued joint damage [2, 7–10, 25, 26]. This report outlines the technical details and clinical case series of patients undergoing subtrochanteric femoral derotational osteotomy for ante- and retrotorsional abnormalities. This technique may be indicated when the intertrochanteric level is not suitable or not any more accessible for derotation, as in hips in which the greater trochanter has to be advanced, relative lengthening of the neck is required and/or osteotomy of the neck or head is executed at the same time. If needed, derotation may be combined with shortening, as in cases with high subluxation or dislocation.

## SURGICAL TECHNIQUE

Physical examination and pre-operative imaging studies should guide options for intra-operative deformity correction. Pre-operative planning should account for the constellation of patient findings, which may require procedures on the femoral and acetabular side, the confluence of surgical incisions and exposure and the coordination of osteotomy planes. Correction goals should be considered in terms of a sequential approach to osteotomy correction [18], and pre-operative planning remains only a guide, which is subject to intra-operative adjustment tailored to the individual patient scenario.

**Fig. 1.**
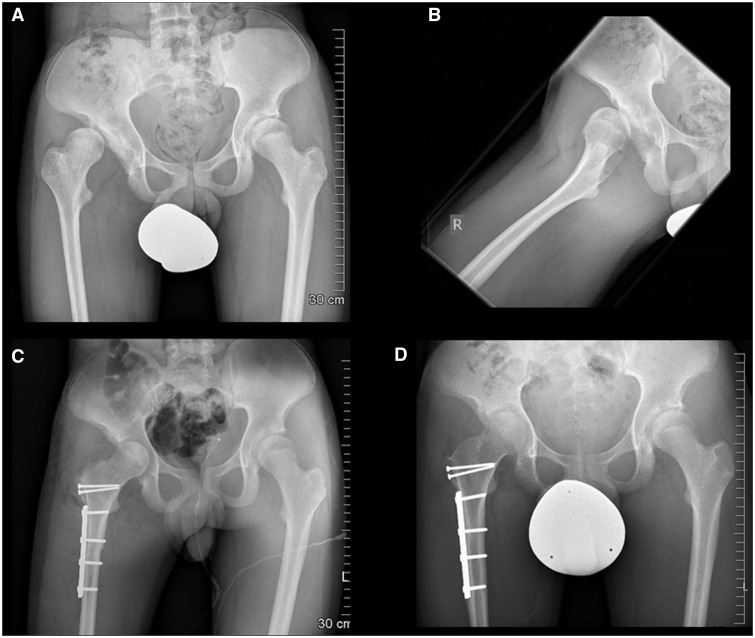
Pre-operative anteroposterior pelvis (A) and right hip frog leg lateral (B) views in a 15-year-old male with Trisomy 21 demonstrating high subluxation. Intraoperative evidence of acetabular cartilage damage was too severe for redirection, but there was good femoral head cartilage. Appropriate reduction of the dislocated head was achieved with capsular interposition arthroplasty, relative neck lengthening and subtrochanteric derotational shortening osteotomy (C). Satisfactory containment was maintained at latest follow-up (D).

**Fig. 2.**
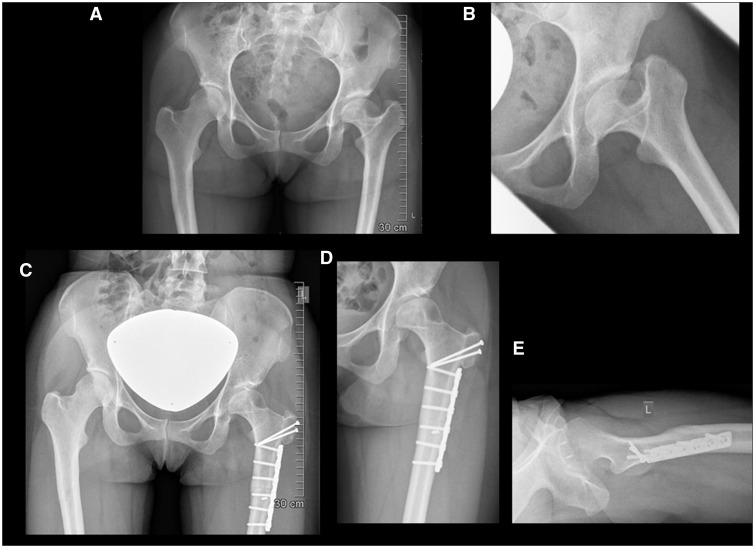
Anteroposterior pelvis (A) projection in a 32-year-old female with Perthes disease, who experienced symptomatic left hip pelvitrochanteric impingement in abduction (B). Post-operative anteroposterior pelvis (C), hip (D) and lateral (E) views showing adequate relative neck lengthening in combination with derotational osteotomy. Intra-articular cam impingement was treated with osteochondroplasty of the anterolateral head-neck junction and labral re-fixation was performed after decompression of a large paralabral ganglion cyst at the area of femoroacetabular conflict. Joint stability was tested while the capsule was still open and the need for a stabilizing periacetabular osteotomy deemed not necessary.

In our experience, the derotational procedure is frequently performed in the setting of an accompanying surgical dislocation of the hip, and these procedures and approaches are complementary. Of note, less than approximately 2% of all surgical dislocations performed at the surgeons’ primary institution involve a concomitant derotation osteotomy. Hip dislocation affords circumferential view of the head, neck and proximal femur, which aids in screw placement, range of motion assessment and maximization of impingement correction. If other procedures (e.g. peri-acetabular osteotomy for acetabular dysplasia) are performed in conjunction with derotation of the femur, we support addressing the femoral-sided deformity first. This femur-first approach [18] is for several reasons: balancing deformity correction with other procedures, such as periacetabular osteotomy or capsular interposition arthroplasty, may be determined finally after intra-operative assessment (Fig. 1). In a complex problem of both acetabular and femoral version issues, after correction of the femoral side, trimming of the acetabular rim may be sufficient to achieve sufficient improvement in range of motion and stability, even if the operative planning may have included acetabular reorientation initially. When the greater trochanter is high riding, it can be an obstacle for accurate acetabular reorientation; therefore, it has to be osteotomized first (Fig. 2). When starting on the femoral side, the first ischial cut of a periacetabular reorientation osteotomy can be performed via this approach under direct visualization and under optimal protection of the sciatic nerve [29]. Finally, changing position of the patient without re-draping is easier from a lateral to supine position.

The patient is placed in the lateral decubitus position on a standard operating table. The entire operative limb is sterilized and draped, allowing for full mobilization of the extremity. A U-shaped foam pillow protects the contralateral limb, and, at the same time, it allows for stable placement of the mobile leg in neutral hip abduction/ adduction.

**Fig. 3.**
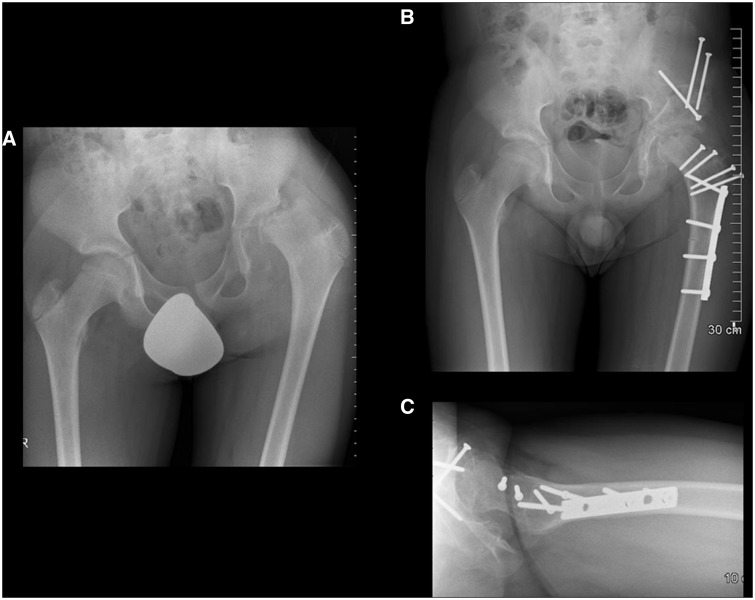
Twelve-year-old male with Perthes disease, severe head extrusion and hinged abduction of the left hip (A) who underwent head-reduction osteotomy, subtrochanteric derotational osteotomy and stabilizing peri-acetabular osteotomy. Post-operative anteroposterior pelvis (A) and lateral hip (B) views demonstrate the advantage of subtrochanteric plating when concomitant multiple fixation screws are required in the femoral neck and greater trochanteric regions.

The procedure starts with the proximal part of the lateral approach for simultaneous surgical dislocation of the hip, as previously described [30]. The hip capsule is exposed, and a capsulotomy is performed. After the indication for an additional periacetabular osteotomy is confirmed, an incomplete cut of the ischial osteotomy is performed now, according to the described technique [29]. Thereafter, the necessary intra-capsular procedures, such as head-neck osteochondroplasty, relative lengthening of the neck, osteotomy of the neck and/ or osteotomy of the head, are executed. Especially in Perthes or Perthes-like hips, the actual amount of pathological version of the femoral neck becomes better visible after relative lengthening of the neck. The amount of eventual shortening of the femur (e.g. in setting of a Colonna capsular arthroplasty to address high subluxation or complete dislocation, to allow relocation of the head in the newly created socket at the level of the paleo-acetabulum and to reduce soft tissue tension) can also best be pre-estimated at this time. In the case of neck or head osteotomy, it becomes obvious that the interference with screws will not allow an intertrochanteric level for the derotation osteotomy. In the case of relative neck lengthening, the reduced size of the intertrochanteric area with semicircular cortical bone loss will compromise the purchase of a blade plate implant used at this level. Therefore, both situations are managed more effectively at the subtrochanteric level (Fig. 3).

**Fig. 4.**
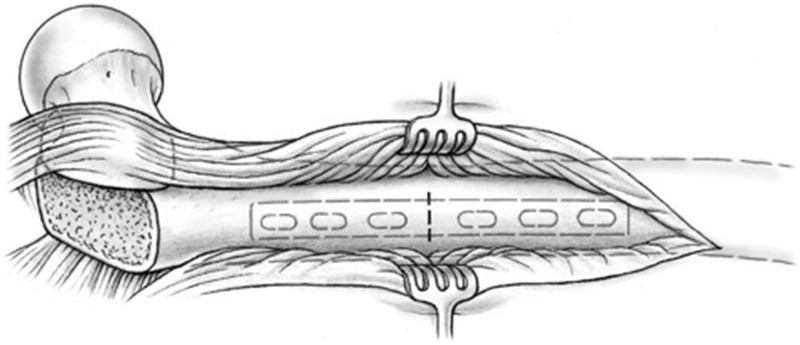
Lateral view of the proximal femur after surgical hip dislocation with greater trochanteric flip osteotomy and distal sub-vastus exposure. The osteotomy site (transverse solid dashed line) is marked with reference to the planned plate position (light gray dashed lines).

**Fig. 5.**
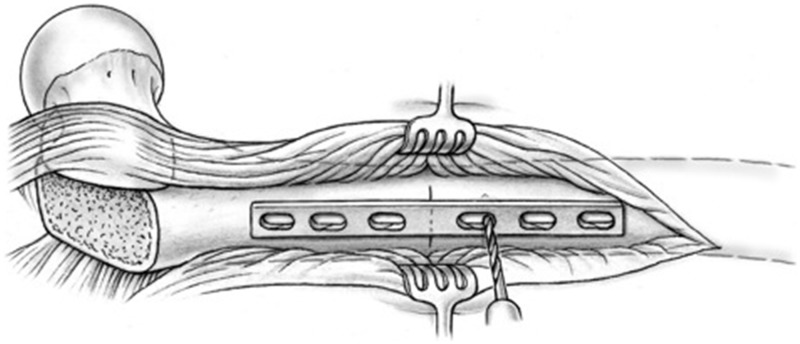
Eccentric drilling of the plate hole closest to the osteotomy site in the distal segment prior to creation of the osteotomy.

**Fig. 6.**
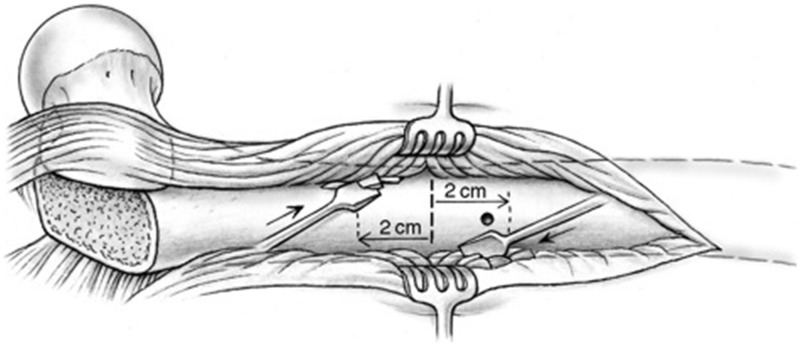
Decortication about the osteotomy site with a sharp osteotome is performed for a length of 2 cm proximal and distal to the location of the osteotomy. The oblique orientation of the osteotome permits the edge of the cutting surface to catch safely a thin slice of bone with attached musculature.

**Fig. 7.**
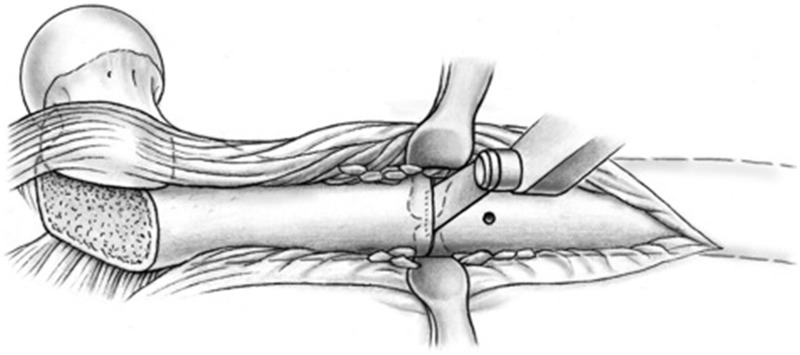
Osteotomy of the femur is made orthogonal to the long axis of the femur, after protection soft tissues with blunt retractors.

A sub-periosteal sleeve proximally along the femur and a distal sub-vastus approach with maintenance of soft-tissue vascularity are performed as needed for adequate exposure. When the necessary distal extension of the approach is performed now and not at the beginning of the procedure, continuous blood loss from a larger wound can be reduced. A non-locking dynamic compression plate is applied to the proximal femur. Because of the ovoid nature of the proximal femur, a mismatch in the cortical thicknesses may arise between the proximal and distal segments after rotation. Therefore, a narrow plate, when compared with the broad option, may carry less risk of rotational displacement of the osteotomy segments due to decreased contact surface area and linear screw array (LC-DCP system). However, plate width and length must be chosen with respect to other patient characteristics, such as body size and particular surface anatomy. Consideration may be made for other broad plating systems that may have linear screw arrays. The osteotomy site is identified with the aid of a provisional six-hole plate (Fig. 4). A first screw hole is drilled in the distal fragment, close to the osteotomy level (Fig. 5) and the plate and screw are removed. Thereafter, a decortication over a distance of about 2 cm proximal and distal to the osteotomy level is performed in the method of Judet [31]. The best instrument for this step is a sharp 10 to 15-mm straight osteotome, which is used as shown in Fig. 6. The result should be a continuous layer of bone chips, with muscle and soft tissue attachments mainly along the anterior and posterior circumference of the diaphysis. An osteotomy orthogonal to the long axis of the femur is performed with a combination of a thin-kerf blade saw and continuous irrigation (Fig. 7). The soft tissues at the osteotomy level are protected with anterior and posterior blunt retractors placed between the decorticated flap and the diaphysis.

**Fig. 8.**
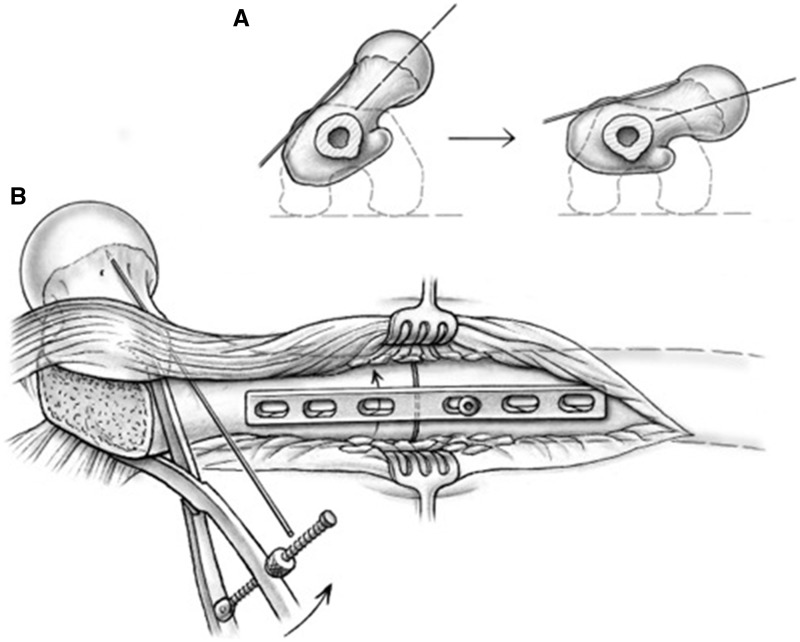
Accurate torsional correction of the proximal femoral segment is accomplished by the circumferential view afforded by the surgical dislocation approach and a supplemental Kirschner wire placed along the axis of the femoral neck. The plate is fixed on the distal segment serving as a splint against gross segment dislocations during the maneuvers. The proximal segment is anteriorly rotated with the help of a Verbrugge clamp (A). The change in the angle between the Kirschner wire and the leg flexed 90° shows the decrease in femoral anteversion (B).

With use of the surgical dislocation technique, the surgeon has complete control of the proximal segment, and preferentially the proximal segment (rather than entire distal limb) is rotated to obtain the desired correction (Fig. 8). First, the plate is fixed again onto the distal segment. The proximal segment, with the Kirschner wire placed on the anterior neck, is then rotated internally for antetorsion correction (rotated externally for retrotorsion correction) with the help of a Verbrugge clamp, whereas the distal segment remains immobile. The plate serves as a splinting aid and particularly avoids medial dislocation of the distal segment during the rotational correction (Fig. 8).

We prefer to determine the degree of correction required through a combined approach: direct visualization of achieved joint stability and removal of femoroacetabular conflict, along with assessment of the overall rotational alignment of the limb. A Kirschner wire placed along the anterior surface of the neck (Fig. 8) allows a rather precise estimation of the version when its direction is compared with the position of the leg in 90° of knee flexion. Optionally, the determined correction can be displayed with two Kirschner wires drilled into the cortex proximal and distal to the osteotomy level; however, these wires often interfere with the correction maneuver and subsequent fixation of the plate. The authors believe that a longitudinal derotation line at the level of osteotomy made with an osteotome or other marking tool lacks precision, as the line may often be covered with the ultimate plate and therefore hard to use as a control at the end for assessing final correction. Although we support the use of any of these intra-operative tools (including angular measurement triangles) based on the particular surgeon’s experience and training, we choose the ultimate correction based on an appropriate anteversion of the femoral neck/proximal fragment while keeping the operative limb (distal fragment) parallel to the long axis of the body. This is confirmed with intra-operative testing of range of motion to ensure optimization of impingement-free range of motion and joint stability.

**Fig. 9.**
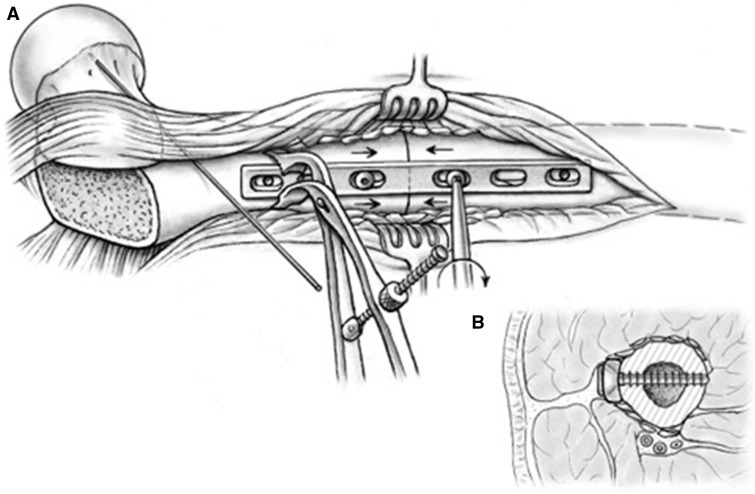
Sequential compression across the osteotomy site is achieved through tightening of the plate screws (A); the degree of reduction is maintained with the assistance of a Verbrugge clamp. Cross-sectional view (B) along the plate demonstrates ideal plate-bone apposition and decortication autograft bone chips enveloped within the soft tissue attachments of the proximal femur.

When the desired rotation is achieved, the segment ends are brought into close contact, and the plate is held against the proximal fragment with an additional Verbrugge clamp. The inserted screw is unfastened and brought in a compression mode position by pushing against the distal end of the plate. The plate is held flush to the lateral bone surface in a way that later screw insertion will not create adverse rotation. The screw hole in the proximal fragment next to the osteotomy is also drilled in compression mode. Both screws are inserted deeper and in an alternating fashion to create compression across the osteotomy (Fig. 9). Achieved rotational correction and correct alignment of the plate over the bone is now checked again, before additional screws are inserted. With good bone quality in a reliable patient, three screws per segment are sufficient; when in doubt, or in poor bone quality, more screws may be inserted. An articulating tensioning device may be applied for supplemental compression as needed. The device may be anchored either distally along the femoral shaft or within the exposed bed of the greater trochanter.

With the joint still exposed, a final check is performed for hip range of motion and achieved torsional correction. Likewise, the hip joint is assessed for appropriate stability, impingement-free range of motion and congruency. Fluoroscopy may be used to document intra-operative osteotomy fixation and joint alignment. The wound is then irrigated and closed in sequential layers, to begin with re-approximation of the capsular flaps and trochanteric re-fixation, followed by repair of the origin of the vastus lateralis muscle.

If not adjusted for additional osteotomies and concomitant procedures, the patient is mobilized with a 15-kg weight restriction (partial weight-bearing) for 6–8 weeks post-operatively. Radiographs may be taken at 6 weeks post-operatively to assess appropriate interval healing. Progression of weight bearing may be delayed to 8 weeks as needed. Other factors, such as age, co-morbidities and body mass index, may play a role in the decision to advance weight bearing. Progression of weight bearing is permitted thereafter, depending on evidence of satisfactory interval radiographic healing. Abductor exercises are allowed at 4–6 weeks with appropriate trochanteric stability.

## PATIENTS AND METHODS

Twenty-eight consecutive subtrochanteric femoral osteotomies performed in 26 patients (21 females and 5 males) were retrospectively reviewed. Patient selection for derotation was based on functional status, physical examination and the degree of abnormal femoral torsion. Standardized torsional imaging was not routinely performed pre-operatively, including for those patients referred to our institution with cross-sectional imaging studies already performed without torsional measurements.

Correction of both antetorsion (*n* = 26) and retrotorsion (*n* = 2) deformities was performed using this technique in this series of patients. There is variation in the definition of femoral mal-torsion [12, 32–35]: for example, Fabricant *et al.* used >25° as threshold for high anteversion ([33]). Jackson *et al.* looked at standard deviations in their cohort and defined retroversion as −2° or less and excessive anteversion as 18° or greater ([34]). The decision to perform a femoral derotation in our study was for values of antetorsion over 20° or less than 0° (retrotorsion) in the setting of femoroacetabular conflict and/or joint instability. Although these parameters guided our definition of femoral mal-torsion, we must emphasize that the final extent of derotation was determined by intra-operative assessment of joint stability and impingement-free range of motion and in accordance with the correction of other deformities. 

Routine post-operative CT scans were not performed on the patients. The amount of correction of high femoral anteversion was on average 15–20° (to achieve a final antetorsion of 10–15°; maximum correction of 40°). In a patient with 3° of pre-operative retrotorsion, a 20° anteversion-producing derotation was performed. Because of the complex nature of this patient group, in general, the corrections were individualized to the particular clinical situation and often considered along with other deformities of the femur and acetabulum. In Perthes cases, pre-operative CT measurements were often inaccurate due to deformity of the head, short neck and bulky trochanteric region; the real amount of torsional deformity became visible only after relative lengthening of the neck. Shortening osteotomy, in conjunction with derotation, was performed in 11 cases. This was performed in the setting of high dislocation.

Underlying diagnoses included dysplasia (Perthes, high and low subluxation/dislocation), arthrogryposis, cerebral palsy, Down’s syndrome, instability and femoroacetabular impingement. Clinical instability was confirmed intra-operatively during testing of the open joint and observation of head migration during range-of-motion.

Prior ipsilateral hip procedures included proximal femoral osteotomy (three patients), intra-articular Chiari osteotomy (one patient), hip arthroscopy (one patient) and unknown (two patients). The mean age at time of surgery was 21.4 years (range, 12–43 years). The two bilateral osteotomies were performed in a staged fashion. There were 14 right-sided procedures and 14 left-sided procedures. All patients underwent concomitant surgical hip dislocation. Outcome measures included serial radiographs and clinical evaluation. Table I

**Table I.** Characteristics of patients undergoing subtrochanteric derotational osteotomy

Procedure	Age (years)	Sex	Side	Concomitant procedures^a^	Subtrochanteric derotational osteotomy fixation	Complication
1	15	Male	Right	Capsular interposition arthroplasty	4.5 mm DCP, 6-hole, narrow	No
2	13	Male	Right	Cyst autografting from trochanter, rim-trimming, dorsal trochanter reduction	4.5 mm DCP, 6-hole, narrow	No
3	27	Female	Right	Labral re-fixation, offset correction, microfracture femoral head, relative HNL	4.5 mm DCP, 6-hole, broad	No
4	12	Male	Left	Head-reduction osteotomy with retinacular flap, PAO	4.5 mm DCP, 6-hole, narrow	No
5	33	Female	Left	Labral re-fixation	4.5 mm DCP, 6-hole, narrow	No
6^b^	25	Male	Left	Labral re-fixation, rim-trimming, acetabular subchondral drilling, offset correction	4.5 mm DCP, 6-hole, broad	No
7^b^	27	Male	Right	Labral re-fixation, rim-trimming, offset correction	4.5 mm DCP, 6-hole, broad	Yes (implant failure)^c^
8	23	Female	Right	Labral re-fixation, rim-trimming, relative HNL	4.5 mm LC-DCP, 7-hole, narrow	No
9	27	Female	Right	Labral re-fixation, acetabular subchondral drilling, offset correction	4.5 mm DCP, 6-hole, narrow	Yes (non-union)^c^
10^d^	33	Female	Right	Capsule revision, rim-trimming, labral re-fixation, offset correction, dorsal trochanter reduction	4.5 mm DCP, 6-hole, narrow	No
11^d^	35	Female	Left	Labral re-fixation, offset correction	4.5 mm DCP, 6-hole, narrow	No
12	43	Female	Left	Offset correction	4.5 mm DCP, 6-hole, narrow	No
13	26	Female	Left	Relative HNL, PAO	4.5 mm DCP, 6-hole, narrow	No
14	22	Female	Left	Relative HNL	4.5 mm DCP, 6-hole, narrow	No
15	22	Female	Right	Relative HNL, SFO, PAO	4.5 mm DCP, 6-hole, narrow	No
16	20	Female	Left	Relative HNL	4.5 mm DCP, 6-hole, narrow	No
17	16	Female	Left	Relative HNL, PAO	4.5 mm DCP, 6-hole, narrow	No
18	25	Male	Right	Relative HNL, capsular arthroplasty, shelf osteotomy, SFO	4.5 mm DCP, 6-hole, narrow	No
19	16	Female	Right	Relative HNL, capsular arthroplasty, SFO	4.5 mm DCP, 6-hole, narrow	No
20	21	Female	Left	Relative HNL, capsular arthroplasty, head reduction osteotomy, SFO	4.5 mm DCP, 6-hole, narrow	No
21	13	Female	Left	Relative HNL, osteoplasty	4.5 mm DCP, 6-hole, narrow	No
22	15	Female	Left	Capsular arthroplasty, SFO	4.5 mm DCP, 6-hole, narrow	No
23	13	Female	Left	Capsular arthroplasty, shelf osteotomy, SFO	4.5 mm DCP, 6-hole, narrow	No
24	20	Female	Right	Relative HNL, capsular arthroplasty, shelf osteotomy, SFO	4.5 mm DCP, 6-hole, narrow	No
25	13	Female	Left	Relative HNL, capsular arthroplasty, shelf osteotomy, SFO	4.5 mm DCP, 6-hole, narrow	No
26	14	Female	Right	Relative HNL, capsular arthroplasty, shelf osteotomy, SFO	4.5 mm DCP, 6-hole, narrow	No
27	18	Female	Right	Relative HNL, capsular arthroplasty, shelf osteotomy, SFO	4.5 mm DCP, 6-hole, narrow	No
28	12	Female	Right	Relative HNL, capsular arthroplasty, shelf osteotomy, SFO	4.5 mm DCP, 6-hole, narrow	No

DCP, dynamic compression plate (Synthes, GmbH, Zuckwil, Switzerland); HNL, head-neck lengthening; PAO, periacetabular osteotomy (Bernese); LC-DCP, limited contact, dynamic compression plate (Synthes, GmbH, Zuckwil, Switzerland); SFO, shortening femoral osteotomy.

^a^All patients underwent surgical dislocation of the hip.

^b^Staged bilateral.

^c^Successful osteotomy union after revision internal fixation.

^d^Staged bilateral.

presents an overview of the cohort.

## RESULTS

All patients, including two patients who required revision surgery, realized successful union by clinical and radiographic analysis at latest follow-up. From radiographic review, one can conclude that full osteotomy healing at this level can be expected by approximately 10–12 weeks (up to 16 weeks), but signs of consolidation may be seen as early as 6 weeks.

**Fig. 10.**
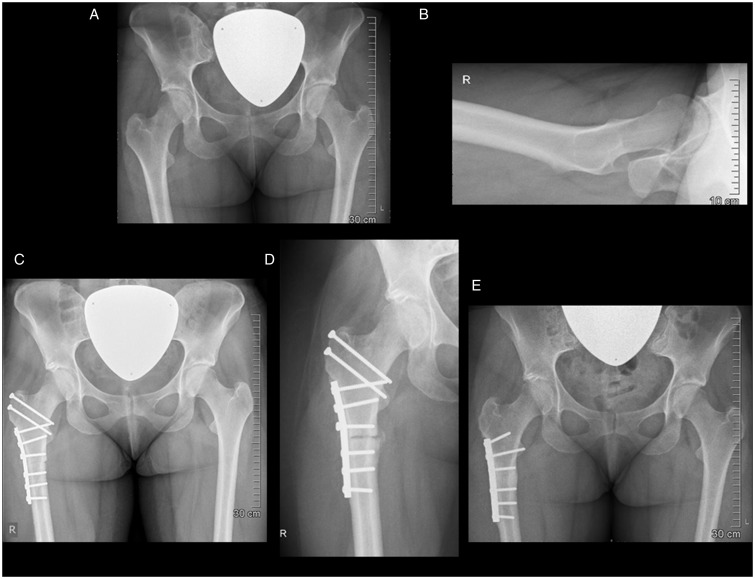
Pre-operative anteroposterior pelvis (A) and right hip lateral (B) views in a 27-year-old female with symptomatic anterior rotatory instability and early degenerative changes. Offset correction and labral re-fixation was performed along with a derotational osteotomy of 15° correction (C). For evidence of poor bony union (D) in the setting of chronic smoking, the patient underwent revision fixation 6 months after index surgery. Satisfactory osteotomy site healing was achieved (E) and, at 2 years of post-operative follow-up, she had a good clinical outcome with no instability observed and resolution of her symptoms.

There were two initial failures for a 7% rate of osteotomy-related complications. One delayed union occurred in a 27-year-old chronic smoker (Fig. 10) with a diagnosis of coxa valga and 40° of femoral neck antetorsion and resulting anterior rotatory instability of the hip. Despite extensive smoking cessation counseling, this patient unfortunately continued to smoke throughout the peri-operative and post-operative period. This prompted revision fixation with a six-hole, broad 4.5-mm plate (index plate was six holes). At 2 years of post-operative follow-up, and after repair of her osteotomy non-union, she had a good clinical and radiographic outcome with no instability observed and resolution of her symptoms. The other failure was attributed to self-accelerated weight bearing in a patient status post successful contralateral derotational osteotomy (contralateral osteotomy was performed 19 months prior to the side that experienced the complication). Revision to a seven-hole, broad 4.5-mm plate (index plate was six holes) was performed, with uneventful healing. One adductor tenotomy unrelated to the subtrochanteric osteotomy fixation was performed in another patient 7 months after index surgery. No greater trochanteric osteotomy site issues were encountered. None of the patients complained about pain of the trochanteric area when lying on the side, a complaint heard frequently from patients treated with a fixed angle blade plate with an angular offset.

## DISCUSSION

Pathologic femoral torsion is a common developmental deformity that affects alignment of the lower limb. Importantly, femoral retrotorsion is known to exacerbate or contribute to hip impingement [36–40], and femoral antetorsion may exacerbate underlying instability [41]. Functional disability and pain may result from untreated femoral torsion abnormalities [2, 7–10]. Evidence from revision hip preservation surgery suggests that joint damage and symptoms are likely to progress if underlying structural factors are not corrected [4, 11, 42–45]. Depending on age and other anatomic abnormalities, derotational osteotomies of the femur may prevent degenerative changes [25, 26].

Although a number of treatment strategies have been described for addressing femoral mal-torsion, including traditional blade-plate application [19, 20], both the location of osteotomy and the type of procedure have implications for effective reconstruction. Various osteotomies in intertrochanteric, subtrochanteric, diaphyseal and supracondylar locations [5, 6, 19, 21–23], and with different fixation techniques and devices, have been described to correct lower extremity rotation [5, 6, 19, 21, 24–28]. The rate of osteotomy-related complications in the literature [23, 25, 27], reported as high as 15% in certain pediatric populations [26], highlights the importance of refined surgical techniques. This report describes our preferred method for addressing femoral torsional abnormalities, with technical considerations for complex hip pathologies, and in patients for whom an intertrochanteric osteotomy is not feasible. A 7% complication rate was observed.

This retrospective review and surgical technique is limited by small patient numbers and no direct comparison to other fixation techniques. No standardized clinical outcome scoring was obtained, as the surgeries were performed in three separate hospital centers and systemic disease (e.g. spastic diplegia, Trisomy 21 and arthrogryposis) might influence standardized outcome scoring. The heterogeneity of underlying diagnoses and concomitant procedures is characteristic of this complex hip population. However, this supports the applicability of this technique across varying hip pathologies. Another limitation is the lack of standardized torsional imaging pre-operatively, including for those patients referred to our institution with cross-sectional imaging studies already performed without torsional measurements, as well as routine CT or magnetic resonance imaging scans for all patients post-operatively. The standardized approach of dynamic compression plating with aid of surgical dislocation also offsets the limits of a small sample size in this challenging patient population. Extension of this technique to more patients will offer insight into whether smoking and early weight-bearing are certain risk factors for early failure, as evidenced by the two initial failures in this series. The two failures have not forced us to consider a modification of technique but rather for better patient selection and more rigorous guidance in the postoperative period. Likewise, obesity and activity level may prove important factors when considering the use of plate size with this subtrochanteric technique. We encourage the application of this procedure in other centers, as well as in prospective study, to further assess the utility in derotational osteotomies for femoral mal-torsion. Further study should more closely inform our understanding of the relationship between femoral torsion abnormalities and the potential for subsequent degenerative changes of the hip; the literature on this causal relationship is certainly limited at this time.

The key advantage of this plate fixation method, in comparison to the traditional technique of fixed-angle blade plating, is proximal fixation that does not interfere with additional procedures such as relative neck lengthening, neck osteotomy and/ or head osteotomy. Moreover, the surgeon can avoid trochanteric complications associated with blade-plate introduction, as well as less hardware profile in the femoral neck. In distinction to the fixation of a blade plate, the greater trochanteric bone, along with the upper femoral epiphysis and growth plate in younger patients (if applicable), is less likely to be violated with this subtrochanteric construct. Likewise, a straight plate can be placed, so that it does not interfere with screw fixation of the greater trochanter when the osteotomy fragment is either re-fixed in its original bed or advanced for adjustment of the abductor lever arm. Dynamic compression plate may be less demanding technically when compared with blade plating. Other alternative techniques, such as external fixation in the method of Ilizarov, are often not as readily accepted in a population of mainly young adults. Finally, subtrochanteric plating does elicit less lateral trochanteric pain and bursitis when compared with a more prominent blade plate. In comparison to osteotomy at or just below the level of the lesser trochanter (or intertrochanteric osteotomy), subtrochanteric fixation requires more distal dissection but obviates the need to include both the derotational osteotomy and greater trochanteric osteotomy re-fixation in a single plating during surgical hip dislocation approach. The accompanying risks of greater trochanter fracture and other complications related to the single fixation method are also reduced.

Healing time of subtrochanteric cortical bone is generally accepted to be longer compared with that of the intertrochanteric cancellous bone. In our series, 10–12 weeks for healing may be compared with 8–10 weeks for that of intertrochanteric osteotomies [46]. Decortication is an established adjunct to accelerate bridging callus, and, as such, the early stability of fixation; the time for complete consolidation of a cortical osteotomy is, however, less affected. Decortication is mainly applied in non-unions of long bones [31] but has a similar effect in osteotomies. In our two failures, the result was not primarily technical but could be explained in one case with a retardation of bone turnover from heavy smoking, and, in the other, better patient compliance could have helped to avoid excessive early overloading of the construct. The plate failure was identified on radiographs 9 weeks after the index surgery (at 6 weeks, the plate was intact but with incomplete osteotomy healing). This plating construct was revised 10 weeks after the index surgery. The non-union case was confirmed at eighteen weeks by serial radiographs (at 6 weeks after the index surgery, there was evidence of incomplete osteotomy healing). This osteotomy was revised 19 weeks after the index surgery. Although osteotomy at the distal femoral metaphysis may have the advantage of faster consolidation compared with a subtrochanteric level, this would comprise loss of direct control of the correction and would require an additional incision.

When compared with another series [47] focusing on similar subtrochanteric dynamic compression plating in a pediatric cohort, our technique does not require significant contouring of the plate to match the proximal femoral anatomy. Moreover, although no mechanical failures were reported in the contrasted series [47], this other technique is unknown in adult populations with higher loading conditions.

Over- or under-correction of femoral torsion can occur [27, 48]. One study [48] reported overcorrection in 2 of 33 osteotomies. Ideal correction of femoral torsion is personalized and not necessarily numeric. It takes into consideration acetabular version, joint stability, as well as impingement-free motion with optional equalized rotation. This is best performed with visualization of the long axis of the femoral neck, acetabular inspection and motion testing with the joint open.

In general, the decision to perform a femoral derotation is for pre-correction values of antetorsion over 20° or less than 0° (retrotorsion). When additional acetabular correction is planned, lower values influencing joint stability or producing posterior impingement may be compensated for on the acetabular side. Retrotorsion can rarely be compensated with additional acetabular correction or anterior head-neck osteochondroplasty alone.

Use of a subtrochanteric derotation osteotomy should be considered in cases in which pathologic femoral mal-torsion contributes to femoroacetabular impingement that is not corrected with intra-articular work alone and/ or additional acetabular correction. The effects of extra-articular torsional correction should be then assessed by intra-articular inspection of the femoroacetabular collisions, with the goal of optimizing the amount of impingement-free range of motion of the limb. Joint stability or coaptation is the other allied goal. Appropriate osteotomy stability is generally achieved with a 4.5 mm broad or small plate according to the size of the femur and the fixation method described.

In conclusion, surgeon awareness of torsional issues, along with versatility in intra-operative techniques and decision making, is paramount. Subtrochanteric plating with this technique offers unique advantages when compared with fixed-angle blade plating, the most important of these being avoidance of trochanteric complications. In the setting of complex joint preservation surgery, this method can be combined with other procedures about the hip, including periacetabular osteotomy and osteotomy of the femoral head and neck. The correction of dynamic torsional abnormalities requires a dynamic intra-operative method of assessment and the ability to balance intra- and extra-articular sources of impingement and/ or instability. Our experience indicates that subtrochanteric osteotomy of the femur using a compression plate and decortication, via a surgical dislocation approach, is a reproducible and stable technique. All anatomic factors leading to hip pain and symptomatic impingement or instability may be addressed, to minimize chondrolabral damage. A femoral osteotomy-first approach to complex deformities of the femur and acetabulum offers the opportunity for sequential correction of co-existing pathologies. Moreover, there exists synergism between this derotational technique and adjunctive surgical dislocation of the hip for torsional correction.

## FUNDING STATEMENT

No funding was utilized for the purposes of this study.
